# Validity of smartwatch-derived estimates of lactate threshold heart rate and pace compared to graded exercise testing

**DOI:** 10.3389/fphys.2025.1621996

**Published:** 2025-07-04

**Authors:** Changda Lu, Wei Cui, Zheng Zhu, Yiwei Wu, Qingjun Xing, Bingyu Pan, Yanfei Shen

**Affiliations:** ^1^ School of Sport Science, Beijing Sport University, Beijing, China; ^2^ School of Sports Engineering, Beijing Sport University, Beijing, China; ^3^ China Sports Big Data Center, Beijing Sport University, Beijing, China; ^4^ Engineering Research Center of Strength and Conditioning Training Key Core Technology Integrated System and Equipment, Ministry of Education, Beijing, China

**Keywords:** exercise intensity, running, lactate threshold, smartwatch, validity

## Abstract

**Background:**

Quantifying exercise intensity through the lactate threshold (LT) is crucial for optimizing athletic training regimes. Traditional methods like maximal lactate steady state and graded exercise testing are valid but invasive and costly. Advances in smartwatch technology offer a non-invasive alternative for monitoring LT, though their measurement protocols and outcomes have been less validated.

**Methods:**

This study evaluates the validity of three mainstream smartwatches (Huawei GT Runner®, Garmin Forerunner 265® or 265s®, and Coros Pace3®) in estimating lactate threshold heart rate (LT HR) and pace (LT Pace), comparing these to measurement protocols and results from the modified Dmax method in laboratory standards. One hundred healthy recreational runners underwent indoor graded exercise tests followed by outdoor tests using Huawei (n = 100), Garmin (n = 23), and Coros (n = 17) smartwatches to compare differences in testing protocols and LT HR and LT Pace.

**Results:**

The success rates for a single test were 78% for Huawei®, 65.22% for Garmin®, and 47.06% for Coros®. For LT HR, no significant differences were observed between smartwatch and DmaxMod estimates across all devices (p > 0.05). The Huawei® watch showed MAE = 10.66 bpm, MAPE = 6.32%; Garmin®: MAE = 11.44 bpm, MAPE = 7.15%; Coros®: MAE = 8.93 bpm, MAPE = 5.95%. Corresponding Pearson correlation coefficients ranged from r = 0.13 to 0.67, and *R* ^2^ values ranged from 0.02 to 0.45. In contrast, LT Pace predictions demonstrated significant overestimation for all devices. Huawei® reported the smallest error (MAE = 1.22 km/h, MAPE = 12.70%, p = 0.01, r = 0.88, *R* ^2^ = 0.78), followed by Garmin® (MAE = 2.17 km/h, MAPE = 25.78%, p < 0.01, r = 0.73, *R* ^2^ = 0.53), and Coros® (MAE = 1.93 km/h, MAPE = 22.63%, p = 0.08, r = 0.79, *R* ^2^ = 0.62). Bland–Altman plots confirmed systematic biases and variable agreement patterns, particularly for LT Pace.

**Conclusion:**

Smartwatches are capable of providing estimates of LT HR and LT Pace in recreational runners, although they tend to overestimate LT Pace and overall accuracy remains to be improved.

## 1 Introduction

Quantifying exercise intensity in endurance sports is essential for enhancing athletic performance (Mujika, 2017). As health awareness increases and activities like running gain popularity, more athletes seek precise methods to monitor and measure their training. Among various quantification techniques, the anaerobic lactate threshold (LT) (Svedahl and MacIntosh, 2003; Faude et al., 2009) is considered the key reference metric for assessing personalized training intensities (Svedahl and MacIntosh, 2003; Heuberger et al., 2018). The LT is characterized by a rapid and distinct change in the slope of the blood lactate concentration curve, indicating the transition from predominantly aerobic to anaerobic energy metabolism (Faude et al., 2009).

In practical training scenarios, lactate threshold heart rate (LT HR) and lactate threshold pace (LT Pace) are commonly used to quantify training intensity. LT HR refers to the heart rate (HR) at the LT (Pfitzinger and Freedson, 1998), while LT Pace refers to the sustainable running speed at this threshold (Yoshida et al., 1987). These indicators are crucial for developing effective and safe training plans, enabling athletes to maintain optimal performance over time, directly affecting their speed and endurance in competitions (Ziogas et al., 2011).

The primary methods for testing LT include the maximal lactate steady state (MLSS) and its more accessible variant, the graded exercise testing (GXT) (Faude et al., 2009; Jones and Ehrsam, 1982; Llodio et al., 2016; Jamnick et al., 2018; Cerezuela-Espejo et al., 2018), which are widely used to determine personalized thresholds for runners (Davis et al., 2007; Antonutto and Di Prampero, 1995; Faude et al., 2009). While these traditional methods are valid (Chalmersa et al., 2024), they are invasive, costly, and rely on specialized equipment and trained personnel (Ramos-Campo et al., 2017; Nascimento et al., 2017; Shiraishi et al., 2018; Di Michele et al., 2012). With the advancement of wearable technology, smartwatches have emerged as integrated solutions incorporating various advanced wearable measurement technologies. These devices, equipped with sensors like GPS, PPG, and IMU, gather kinematic and physiological data, offering non-invasive means to predict a runner’s LT. This technological progress significantly enhances the implementation of training plans (Siepmann and Kowalczuk, 2021; Paul et al., 2024), the monitoring of training sessions (Jubair and Mehenaz, 2024), and fatigue recovery (Jubair and Mehenaz, 2024). However, despite the convenience provided by smartwatches, the validity of their measurements has not been widely validated (Seshadri et al., 2019). Although numerous studies have explored the validity of smartwatches in measuring steps (Gaz et al., 2018; Sears et al., 2017; Veerabhadrappa et al., 2018), energy expenditure (Le et al., 2022; Lee et al., 2018; Nuss et al., 2019), HR (Fuller et al., 2020; Gillinov et al., 2017; Støve et al., 2019; Boolani et al., 2019) and maximum oxygen uptake (VO_2_max) (Carrier et al., 2023; Carrier et al., 2025; Caserman et al., 2024; Jamieson et al., 2024; Muthusamy et al., 2021; Cooper and Shafer, 2019; Kraft and Roberts, 2017), but research on their accuracy in estimating LTs is comparatively limited. Among the limited studies, Schlie observed that the Garmin Fenix 7® tends to underestimate LT HR and LT Pace (Schlie et al., 2024), a finding echoed by Carrier’s research, which also reported an underestimation of LT performance by the Garmin Fenix 6®(Carrier et al., 2021). Additionally, few studies have investigated how the testing protocols of these watches might influence the results. Therefore, this study aims to compare the LT measurement protocols and their results from three mainstream smartwatches against LTs determined using the modified Dmax method (DmaxMod) in the laboratory setting (Siahkouhian et al., 2012), assessing the validity of these wearable devices in terms of their measurement protocols and outcomes for LTs.

## 2 Materials and methods

### 2.1 Participants

This study recruited 100 healthy recreational runners aged 18–65 years as participants. All participants had no history of acute or chronic heart disease, hypertension, joint or musculoskeletal disorders, acute illnesses, pregnancy, or suspected pregnancy. They also had no lower limb injuries or surgeries in the past year. The research protocol adhered to the ethical principles of the Declaration of Helsinki and was approved by the Sports Science Experimental Ethics Committee of Beijing Sport University (2024136H). Before the experiment, all participants were fully informed about the procedures and provided their written informed consent. The consent form confirmed the participants’ voluntary participation, understanding of the study protocol, and right to withdraw from the experiment at any time.

### 2.2 Experimental equipment

• Treadmill: Technogym Excite Run 700®, Technogym.
• Blood Lactate Analyzer: Biosen C-line®, EKF®.
• Heart Rate Monitor Strap: POLAR H10®, POLAR Electro Oy.
• Huawei Watch: HUAWEI GT Runner®, version HarmonyOS 4.0, Huawei Terminal Co., Ltd.
• Garmin Watches: Garmin Forerunner 265® (used for male participants) and Garmin Forerunner 265s® (used for female participants), version 19.18 Garmin International Inc. These two models are functionally identical, differing only in case size and strap length.
• Coros Watch: COROS Pace 3®, version v3.0309, Coros Sports Technology Co., Ltd.

### 2.3 Study design

**FIGURE 1:**
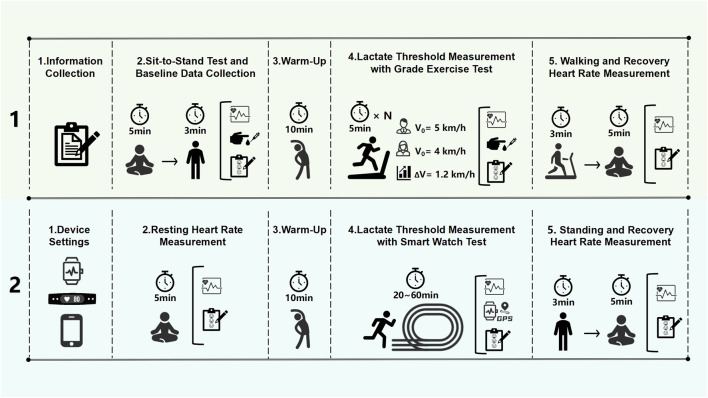
Experimental procedures for lactate threshold heart rate (LT HR) and lactate threshold pace (LT Pace) measurement protocols.

The study was conducted in two phases: an indoor GXT and an outdoor test using smartwatches to assess LT levels. The testing process is depicted in Figure 1. Participants could engage in the outdoor test with one to three smartwatches. A minimum interval of 48 h was maintained between the indoor and outdoor tests and the various outdoor tests to ensure sufficient recovery and accuracy.

### 2.4 Indoor graded exercise test

Researchers collected baseline demographic data, including the participants’ age, height, and weight. Before the exercise test commenced, participants completed a sit-to-stand test, which involved sitting quietly for 5 minutes and standing for 3 minutes; HR measured during this period was recorded as resting HR. Immediately after the sit-to-stand test, fingertip blood samples were taken to assess resting blood lactate levels. Participants then performed a 10-min dynamic warm-up routine by following a standardized instructional video provided by the researcher. Following the warm-up, the treadmill test began at an initial speed of 5 km/h for males and 4 km/h for females, with a 1% incline to offset the external wind resistance encountered outdoors (Davies, 1980). Each stage of the test lasted 5 minutes, followed by a 30-s pause for collecting fingertip blood samples and assessing fatigue using Borg’s Rating of Perceived Exertion (RPE), which ranges from zero (no exhaustion) to ten (maximum exhaustion). The treadmill speed was then increased by 1.2 km/h for the next stage, continuing until the participant reached exhaustion or could not proceed further. Upon completion of the test, participants strolled for 3 minutes, followed by 5 minutes of seated rest, during which recovery HR was measured and RPE scores were reassessed.

### 2.5 Outdoor lactate threshold test with smartwatches

After completing the indoor graded exercise test, participants were informed of the outdoor testing procedures and duration for each smartwatch model. The selection of which smartwatch to test was made voluntarily by each participant based on personal preference and time availability. Participants were allowed to take part in tests for multiple smartwatch models, but a minimum interval of 48 h was required between each test. All outdoor tests were conducted on a standardized 400-m track under non-extreme weather conditions. Testing sessions were scheduled according to participant availability and were typically aligned with their usual training days. All indoor and outdoor tests were scheduled during morning or early afternoon hours to minimize potential circadian effects.

Researchers reset the data on each smartwatch and entered basic participant information such as gender, age, height, and weight into the respective software of each device. Participants were then fitted with the HR strap and the appropriate smartwatch. The smartwatch was placed on the dorsal side of the non-dominant wrist by trained researchers. To ensure consistent data collection conditions, all participants wore a Polar H10 chest strap during testing. For the Garmin® watch, the chest strap was a mandatory requirement to initiate its LT test. For the Huawei® and Coros® watches, the strap was not required for testing. It was connected via Bluetooth to a smartphone with GPS manually disabled, which was used solely forHR logging. The actual HR data for analysis was collected from the watches’ built-in optical PPG sensors. Following this setup, they sat quietly for 5 min to measure their resting HR and assess their initial level of physical and psychological fatigue using Borg’s RPE scale. Subsequently, participants completed a standardized 10-min dynamic warm-up routine via a pre-recorded instructional video. After the warm-up, they ran for 20–60 min, following the HR and pace instructions for each smartwatch’s testing protocol. Throughout the run, all watches guided voice prompts and screen displays to maintain the stability of the HR and pace as much as possible. Upon completion of the run, researchers immediately recorded the participants’ RPE scores.

#### 2.5.1 Huawei® watch testing protocol

The Huawei watch testing required setting the participant’s age and maximum heart rate (HRmax was determined using the peak HR obtained during the indoor test) before the test. The test included an 8-min warm-up phase, followed by gradual increases in HR every 3–5 min. The test was divided into six stages, lasting approximately 20–30 min. Participants were required to complete at least four stages.

#### 2.5.2 Garmin® watch testing protocol

The Garmin® watch test required participants to perform an outdoor run to determine their VO_2_max before initiating the LT test. Employing the Firstbeat® technology, the watch estimated the LT HR and LT Pace by analyzing changes in HR and respiratory rate (which affect heart rate variability). The test included a 10-min warm-up, then progressively increased the HR zones over six stages, each 3–4 min long, totaling approximately 25–30 min.

#### 2.5.3 Coros® watch testing protocol

The Coros watch test required participants to input their 10-km run time. The test commenced with a 5-min warm-up, followed by 25 min of running at a marathon pace, then 3 min at a 10 km pace, and 3 min at a 5 km pace. If this point successfully captured the LT data, the test transitioned to a cool-down phase; if not, one to two additional sets at increased speeds were required. The test included up to 7 phases and lasted approximately 30–60 min. Participants were required to complete at least three running stages.

### 2.6 Determination of the lactate threshold

**FIGURE 2:**
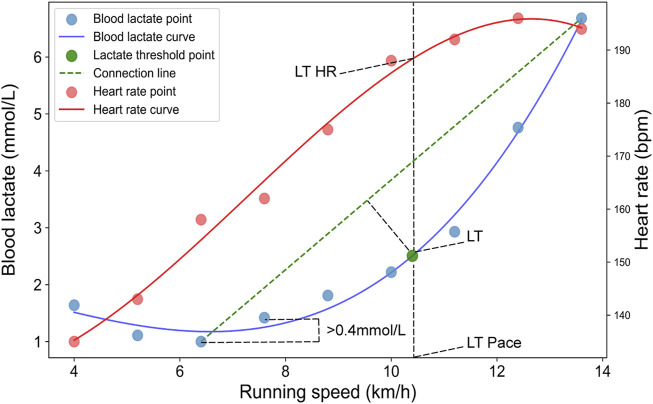
Determination of the lactate threshold using the modified Dmax (DmaxMod) method. Blue dots represent blood lactate concentration (BLC) sampling points, and red dots represent HR sampling points. The blue and red solid lines show the fitted curves for BLC and HR, respectively. The green dot indicates the identified lactate threshold point. The green dashed line connects the first and last BLC data points used to determine the threshold.

The LT of the indoor GXT was calculated using the DmaxMod method (Fabre et al., 2010). Blood lactate concentrations at each stage were interpolated using cubic spline interpolation to generate a smooth and standardized curve for subsequent analysis. A third-order polynomial was then applied to fit the curve of blood lactate concentration as a function of running speed (Zwingmann et al., 2019). The LT was determined as the point on the blood lactate curve that maximizes the distance to the line connecting the first data point where the lactate concentration increases by more than 0.4 mmol/L to the last sample point. The heart rate and running speed at the LT are determined as the values corresponding to the point, as shown in Figure 2. The LT HR and LT Pace for each smartwatch were obtained from data downloaded from the cloud following the successful completion of the outdoor test.

### 2.7 Statistical analysis

Statistical analyses were conducted using Excel (Microsoft, 2021), SPSS version 22 (IBM Statistics, 2021), OriginPro (OriginLab, 2021), and Python version 3.11 to evaluate the predictive accuracy and measurement success rates of three smartwatches in estimating LT HR and LT Pace. Success rates were calculated for each device, and histograms were used to analyze the distribution of results across different age groups and LTs. Data normality was assessed by the Shapiro–Wilk test. For normally distributed variables, independent-samples t-tests were used to compare values between participants with successful and failed tests. The mean absolute error (MAE) and mean absolute percentage error (MAPE) between smartwatch estimates and DmaxMod results were calculated for each device, and Welch’s t-tests were used to determine whether these differences were statistically significant. For variables that did not meet normality assumptions, the Mann-Whitney U test was applied as a nonparametric alternative. Line plots were employed to depict deviations and trends between the estimates provided by the devices and the measurements obtained using the DmaxMod method. Linear regression analyses were executed to plot estimates from the devices against measurements using the DmaxMod method. The coefficient of determination (*R* ^2^) was used to evaluate the model fit, and the Pearson correlation coefficient assessed the strength of the correlation. Bland-Altman plots were used to analyze the agreement and consistency between the estimates from the smartwatches and those obtained using the DmaxMod method, emphasizing the error range and the reliability of the estimation methods employed by the smartwatches. In addition, the two one-sided tests (TOST) procedure was employed to assess the statistical equivalence of LT HR and LT Pace values between smartwatch estimates and DmaxMod measurements. Equivalence bounds were defined using Cohen’s d = ±0.5, with pooled standard deviations used to derive absolute margins.

## 3 Results

### 3.1 Descriptive statistics

This study measured the LTs of 100 participants through an indoor GXT. All participants took part in the outdoor running test using the Huawei® smartwatch. 23 participants engaged in the Garmin® smartwatch test, and 17 tested with the Coros smartwatch. Among all participants, 71 completed outdoor testing with one smartwatch, 17 with two smartwatches, and 11 with all three smartwatches. The basic information of the participants is shown in Table 1.

**TABLE 1:** Basic information of participants.

Watch	Gender ratio (male: Female)	Age (years)	Height (cm)	Weight (kg)
Huawei	61:39	28.0 ± 9.6	170.79 ± 8.34	64.30 ± 11.29
Garmin	13:10	29.13 ± 9.93	170.78 ± 9.58	64.96 ± 11.45
Coros	12:5	25.88 ± 7.11	173.35 ± 6.70	63.26 ± 10.46

### 3.2 Measurement success rates

Since the LT in indoor tests was determined using the DmaxMod method, successful threshold identification was guaranteed as long as data acquisition was completed. In contrast, during the outdoor LT testing using smartwatches, there were instances in which participants completed all testing procedures, but the devices failed to generate LT HR or LT Pace values due to algorithmic limitations. Therefore, we analyzed the success rates of each smartwatch, as well as the distribution of successful and failed tests across different age groups, LT HR, and LT Pace. According to Table 2, Huawei® watches exhibited a higher success rate of 78%, while Garmin® watches showed a moderate success rate of 65.22%. The Coros® watch recorded a lower success rate at only 47.06%. These varied outcomes are likely influenced by the different testing protocols and the complexities associated with each device.

**TABLE 2:** Measurement success rate by smartwatches.

Indicator	Huawei	Garmin	Coros
Total	100	23	17
Successful	78	15	8
Failed	22	8	9
Success rate	78%	65.22%	47.06%

Table 3 shows the distribution of participants with successful and failed tests. Based on the results of Welch’s t-test, we observed no significant differences between the successful and failed test groups However, for the Coros® watch, statistically significant differences were found in both LT HR and LT Pace (p < 0.01), indicating that participants who failed the test tended to have lower LT HR and slower LT Pace compared to those with successful results.

**TABLE 3:** Test outcomes by smartwatches.

Indicator	Group	Huawei	P	Garmin	p	Coros	p
Age (years)	Successful tests	28.47 ± 9.53	0.22	30.93 ± 10.38	0.12	23.88 ± 3.56	0.47
	Failed tests	27.05 ± 10.08		25.75 ± 8.61		27.67 ± 9.07	
LT HR (bpm)	Successful tests	165.12 ± 15.11	0.46	162.14 ± 14.80	0.67	167.45 ± 12.50	0.74
	Failed tests	163.33 ± 16.95		155.66 ± 23.78		159.10 ± 24.38	
LT Pace (km/h)	Successful tests	9.75 ± 2.28	0.31	8.93 ± 1.63	0.46	9.75 ± 2.12	0.36
	Failed tests	9.29 ± 2.86		9.53 ± 2.00		8.94 ± 1.81	

**FIGURE 3:**
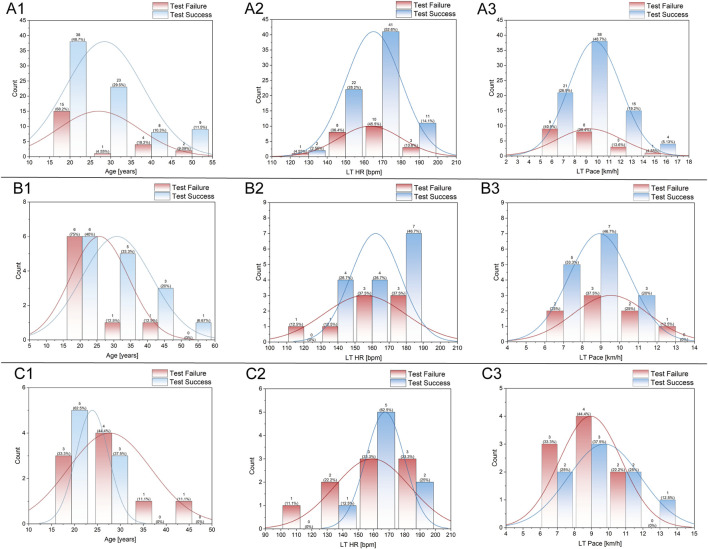
Distribution analysis of participant age **(A1–C1)**, LT HR **(A2–C2)**, and LT Pace **(A3–C3)** in the tests of different smartwatches. **(A1–A3)** represent data from Huawei watches, **(B1–B3)** from Garmin watches, and **(C1–C3)** from COROS watches. Each plot displays histogram distributions for test success (blue) and test failure (red), with overlaid density curves and count annotations for each bin.

Figures 3A1–A3 show that the successful tests of the Huawei® watch primarily occurred in participants below 30 years of age, with an LT HR of approximately 170 bpm and an LT Pace of around 10–11 km/h. Failed tests were predominantly observed in participants with lower LT HR and LT Pace. Figures 3B1–B3 displayed a moderate success rate across a wide range of ages and HR, particularly achieving higher success rates in participants aged 20 to 35, with LT HR between 160–180 bpm and LT Pace of 9–11 km/h. However, there was a higher rate of test failures among the age groups under 20 and those with lower LT HR and LT Pace. As depicted in Figures 3C1–C3, the Coros® watch’s successful tests were mainly concentrated among participants aged 18 to 30, with LT HR around 160–170 bpm and LT Pace primarily around 9–11 km/h. Despite these successes, the overall success rate was relatively low, mainly due to a higher failure rate at atypical LT HR and LT Pace, similar to the other smartwatches.

### 3.3 Predictive accuracy

In this study, we evaluated the prediction accuracy of the Huawei®, Garmin®, and Coros® smartwatches in terms of LT HR and LT Pace by comparing the results of these devices in an outdoor test with the results of an indoor GXT. Regarding LT HR prediction, as shown in Table 4, despite variations in the sample sizes tested by each watch, estimations from all three devices showed no significant differences (p > 0.05) with the results from the DmaxMod method and maintained MAE ranged from 8.93 to 11.44 bpm, and the MAPE ranged from 5.95% to 7.15%. Regarding LT Pace prediction, all devices tended to overestimate LT Pace compared to the DmaxMod method. The Huawei® watch showed the smallest error (MAE = 1.22 km/h, MAPE = 12.70%, p = 0.01), followed by the Garmin® watch (MAE = 2.17 km/h, MAPE = 25.78%, p < 0.01), and the Coros® watch (MAE = 1.93 km/h, MAPE = 22.63%, p = 0.08).

**TABLE 4:** Predictive accuracy of lactate threshold heart rate and lactate threshold pace.

Watch	Type	LT HR (bpm)	MAE (bpm)	MAPE (%)	p	LT pace (km/h)	MAE (km/h)	MAPE (%)	p
Huawei	DmaxMod	169.20 ± 15.12	10.13	6.63%	0.24	9.76 ± 2.28	1.22	12.70%	0.01
	Watch test	167.60 ± 9.93				10.71 ± 2.19			
Garmin	DmaxMod	162.14 ± 14.81	8.93	5.95%	0.15	8.93 ± 1.63	2.17	25.78%	0.00
	Watch test	168.67 ± 8.25				10.99 ± 1.60			
Coros	DmaxMod	167.46 ± 12.50	11.44	7.15%	0.13	9.75 ± 2.12	1.93	22.63%	0.08
	Watch test	175.38 ± 5.18				11.69 ± 2.01			

**FIGURE 4:**
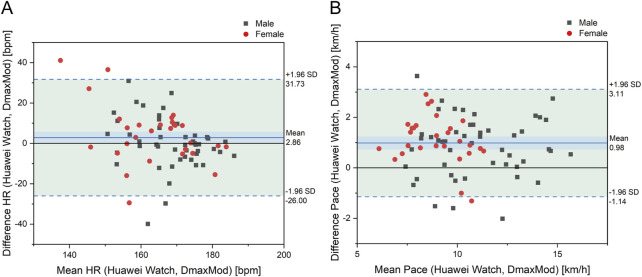
Bland-Altman plot for differences in **(A)** LT HR and **(B)** LT pace between Huawei watch and DmaxMod method.

Figure 4 shows that most data points for both LT HR and LT Pace measured by the Huawei® Watch fall within the ±1.96 standard deviation range from the DmaxMod method reference. The mean deviation is 2.86 bpm for LT HR and 0.98 km/h for LT Pace.

**FIGURE 5:**
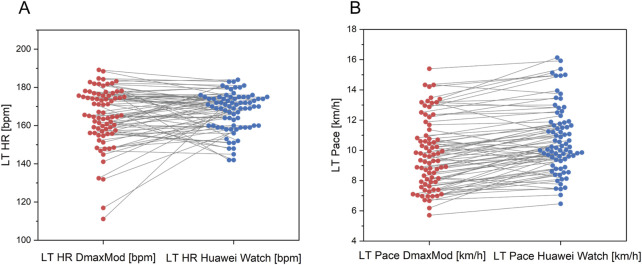
Comparison of **(A)** LT HR and **(B)** LT pace between Huawei watch and DmaxMod method.

Figure 5 illustrates that, compared to the results of the DmaxMod method, the LT HR measurements from the Huawei® Watch are generally more concentrated. There is an underestimation at higher LT HR values and occasional overestimation in some subjects at lower LT HR levels. The distribution of LT Pace measurements is uniform, though it generally trends slightly above the DmaxMod method results, indicating a minor overestimation in Huawei® watch’s LT Pace estimations.

**FIGURE 6:**
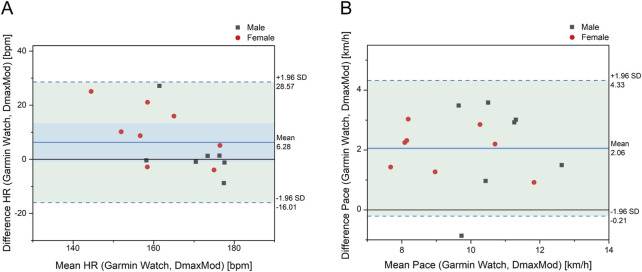
Bland-Altman plot for differences in **(A)** LT HR and LT **(B)** pace between Garmin watch and DmaxMod method.

Figure 6 shows that the prediction of LT HR showed an overestimation, with a mean deviation of 6.28 bpm and a distribution of data points within ±1.96SD, but with a significant degree of dispersion, especially in the low HR interval, showing a considerable deviation. Regarding LT Pace, the mean deviation was 2.06 km/h, with a relatively dispersed distribution of data points, and most of them were located in the positive deviation range.

**FIGURE 7:**
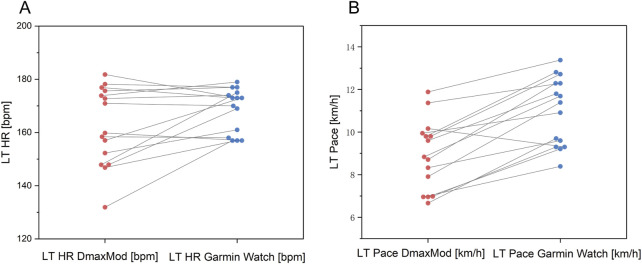
Comparison of **(A)** LT HR and **(B)** LT pace between Garmin watch and DmaxMod method.

Figure 7 shows that the Gamin Watch exhibited underestimation in LT HR prediction, particularly in subjects with lower LT HR. Regarding LT Pace, the prediction results of the Gamin Watch were higher than the results of the DmaxMod method overall.

**FIGURE 8:**
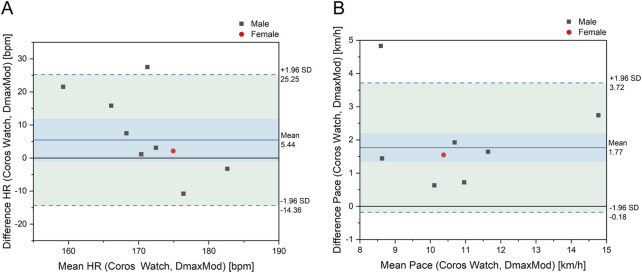
Bland-Altman plot for differences in **(A)** LT HR and **(B)** LT pace between Coros watch and DmaxMod method.

As shown in Figure 8, the mean deviation of LT HR was 5.44 bpm. In contrast, the mean deviation of LT Pace was 1.77 km/h, with the data points in the positive deviation range showing a tendency of overestimation.

**FIGURE 9:**
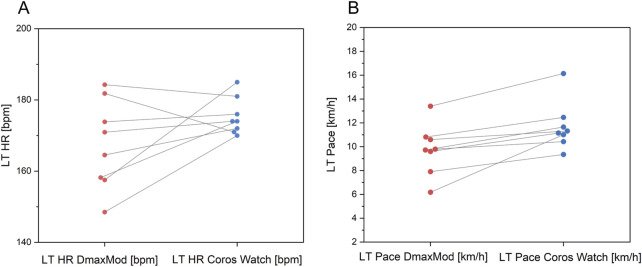
Comparison of LT **(A)** HR and **(B)** LT pace between Coros watch and DmaxMod method.

As shown in Figure 9, the distribution of data points of LT HR of the Coros® watch is concentrated, showing a tendency of overestimation in individuals with lower LT HR and underestimation in those with higher LT HR. As for LT Pace, the prediction results of the Coros® watch are significantly higher than the results of the DmaxMod method, and the deviation of some data points is more significant, reflecting the systematic overestimation of pace prediction by the Coros® watch.

**FIGURE 10:**
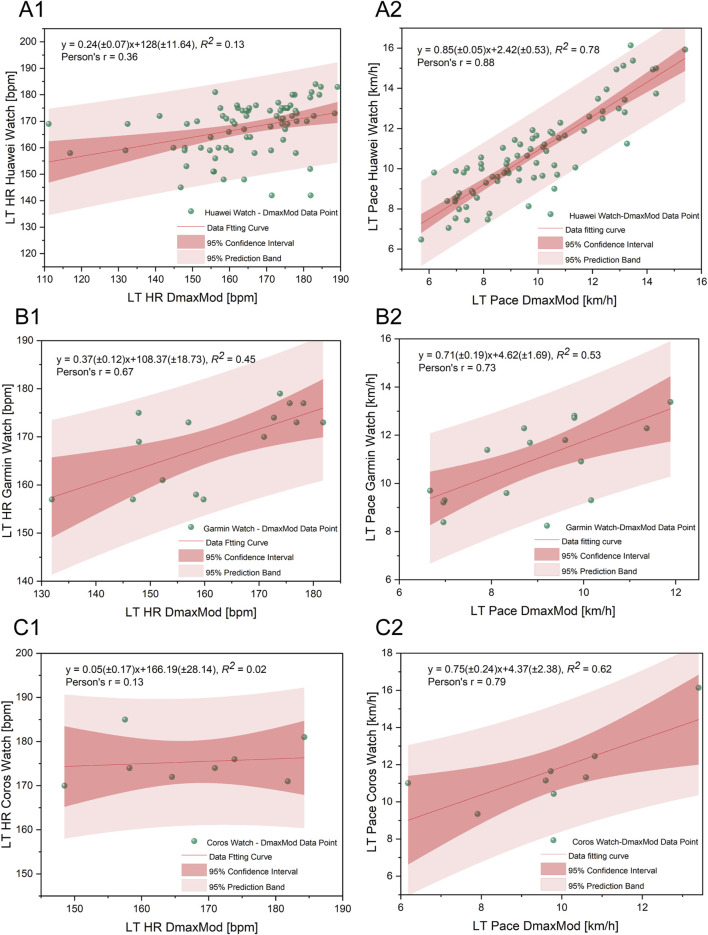
Regression analysis comparing LT HR **(A1–C1)** and LT Pace **(A2–C2)** measured by smartwatches with those calculated using the DmaxMod method. **(A1,A2)** represent data from Huawei watches, **(B1,B2)** from Garmin watches, and **(C1,C2)** from COROS watches. Each plot includes the fitted regression line, 95% confidence interval (dark red band), and 95% prediction band (light red band), with green dots representing individual data points.

As shown in Figure 10 A1 and A1, the Huawei® Watch model for predicting LT HR has a coefficient of determination (*R* ^2^ = 0.13) and a Pearson correlation coefficient (r = 0.36). For LT pace, the coefficients are *R* ^2^ = 0.78 and r = 0.88. As shown in Figure 10B1,B2, the Garmin® Watch model shows a coefficient of determination (*R* ^2^ = 0.45) and a Pearson correlation coefficient (r = 0.67) for LT HR estimations. For LT Pace estimations, the coefficients are *R* ^2^ = 0.53 and r = 0.73. As shown in Figure 12C1,C2, the Coros® Watch model for predicting LT HR has *R* ^2^ = 0.02 and r = 0.13 coefficients. For LT Pace, the coefficients are *R* ^2^ = 0.62 and r = 0.79.

The TOST results indicated that none of the three devices met the equivalence criteria for LT HR. The mean differences were −2.44 bpm for Huawei®, −2.65 bpm for Coros®, and −6.52 bpm for Garmin®. Similarly, for LT Pace, the mean differences were −0.95 km/h for Huawei®, −1.53 km/h for Coros®, and −2.06 km/h for Garmin®. These results suggest that although average deviations were modest in some cases, none of the smartwatch-based estimates could be statistically considered equivalent to laboratory-derived reference values under the predefined thresholds.

## 4 Discussion

### 4.1 Test protocols of each smartwatch

The basic principle of threshold tests is to gradually increase the external load on the runner, which causes a corresponding change in the internal load, and then to monitor the threshold at which significant changes in the runner’s physiological and biochemical parameters occur (Mader and Heck, 1986; Poole et al., 2021). Depending on the indicators used, these tests can be categorized into ventilatory threshold (Hollmann, 2001), LT (Poole et al., 2021; Faude et al., 2009), and heart rate variability threshold (Cottin et al., 2006; Karapetian et al., 2012; Queiroz et al., 2018). The differences in testing methods primarily lie in three areas: 1) the manner in which external load is imposed, 2) the amount and granularity of these loads, and 3) the algorithm used to detect changes in internal load indicators. The LT measurement technologies used in smartwatches varies, but the main differences also fall within the abovementioned aspects. Because manufacturers have not disclosed detailed information about their detection algorithms, this discussion will instead concentrate on the methods of imposing external loads and the amount and granularity of these loads.

### 4.2 Methods of imposing external load

When using smartwatches for LT measurement, hardware capabilities and software interactions limit the methods of imposing external load (Seshadri et al., 2019). Although external load in running tests typically appears as changes in speed, smartwatches cannot directly control a runner’s speed but guide adjustments through prompts. Different prompt methods can directly affect the validity of the test. For instance, the Coros® watch test instructs participants to increase their pace. This approach is straightforward and similar to an indoor GXT (Faude et al., 2009; Jones and Ehrsam, 1982; Llodio et al., 2016; Jamnick et al., 2018; Cerezuela-Espejo et al., 2018). However, the issue with this method is that, although the pace is stable during a test stage, achieving a steady state in HR within about 3 min is challenging (Bailey and Ratcliffe, 1995; Zakynthinaki, 2015; Marini et al., 2022). This increases the difficulty of subsequent detection algorithms. Indeed, due to these reasons, the Coros® watch’s test failure rate is comparatively higher. On the other hand, Huawei® and Garmin® watches employ a different strategy by asking runners to maintain a specific HR range. This subtler method does not require runners to keep a certain pace but enables them to manage their HR by adjusting their running pose. Since the heart muscle is involuntary, runners cannot control their HR directly. It allows runners to maintain it by focusing on their running posture and adjusting their breathing and other physiological processes (Meir et al., 2014; Gu et al., 2017). Therefore, using HR as the control variable may be a more optimal choice, as it can better maintain physiological stability throughout each phase and reduce the complexity of the detection algorithm (Hoppe et al., 2018; Wallace et al., 2014).

### 4.3 Amount and granularity of external load

The amount and granularity of the external load applied in LT tests are influenced by the input indicators provided before the test. For Huawei® watches, the input data can be either the runner’s HRmax or their age. If age is input, the HRmax is calculated using the “220-age.” Garmin® watches use the VO_2_max predicted during outdoor running tests to set parameters. For Coros® watches, the input is based on the runner’s 10-km running performance. Each of these inputs has its limitations. For inputting HRmax, without laboratory measurements, it is difficult for runners to determine their actual HRmax accurately (Abt et al., 2018), and reliance on age for estimation is inaccurate (Shookster et al., 2020; Robergs and Landwehr, 2002). The problem with inputting VO_2_max is similar to that of HRmax. The Garmin® watches do not support manual input, but are measured only through outdoor running tests. Research has shown a discrepancy between the maximum oxygen uptake measured by watches and that measured in laboratories (Molina-Garcia et al., 2022; Kraft and Roberts, 2017; Snyder et al., 2021). This discrepancy can significantly impact the accuracy of the test. When the input indicator is a 10-km performance, runners often enter their best past 10-km time, which could lead to higher pacing in various test phases, potentially increasing the difficulty of the test. Additionally, when considering the input indicators themselves, the stability of a 10-km performance is significantly lower than that of physiological indicators such as HRmax (Düking et al., 2022) and VO_2_max (Molina-Garcia et al., 2022). This can affect the reliability and accuracy of the tests.

### 4.4 Validity of the measurements

In this study, the three smartwatches demonstrated high validity in estimating LT HR, with no significant differences from the results obtained using the DmaxMod method and MAPE ranging between 5.95% and 7.15%. These findings are consistent with those of Carrier et al., who reported a 6.20% error in HR measurements for the Garmin Fenix 6® (Carrier et al., 2021). However, they are slightly higher than the 1.71% error reported by Schlie’s study using the Garmin Fenix 7® during outdoor tests (Schlie et al., 2024). Although the MAPE was low, the MAE of 8.93–11.44 bpm may still affect the precision of training zone determination. Therefore, runners are advised to interpret smartwatch-estimated LT HR in conjunction with perceived exertion to better guide endurance training.

Moreover, the data points from the three watches were quite concentrated, tending to overestimate the values for individuals with lower LT HR and underestimate those with higher LT HR, unlike the DmaxMod method, which can more accurately differentiate HR in marginal zones. This tendency toward that smartwatch algorithms tend to perform best when predicting values near the population average, but may produce less accurate estimations for individuals at the lower or upper extremes of fitness. Such a limitation could reduce the utility of these devices for high-level athletes or individuals with unusually low or high thresholds.

In estimating LT Pace, all three watches demonstrated significant deviations from the DmaxMod results, with MAPE ranging from 12.70% to 25.63%. This finding contrasts with Schlie’s results using the Garmin Fenix 7 in outdoor tests, where the watch slightly underestimated LT Pace (Schlie et al., 2024). This discrepancy may stem from the differences in testing environments. Schlie’s measurements were taken on an outdoor track, while this study was conducted on a treadmill within a laboratory setting. Previous evidence suggests that treadmill running tends to elicit lower blood lactate concentrations at submaximal speeds. And a subtle mismatch between actual pacing and perceived exertion has been reported, indicating that treadmill-based pacing may feel different to participants despite similar physiological responses (Miller et al., 2019).

Additionally, the study found that although the three smartwatches had a high accuracy in predicting LT HR, their regression analysis performance was poor (*R* ^2^ = 0.02–0.45, r = 0.13–0.67) and notably wide limits of agreement. This phenomenon suggests that the high accuracy might not all stem from the validity of the predictive algorithms in the watches but rather from the concentration of different LT HR data distributions, potentially overlooking individual differences among runners. Moreover, this could be influenced by smartwatch manufacturers’ strategies, as showing higher LT HR may push training intensities beyond what runners can comfortably sustain. The conservative approach likely aims to ensure safety. For LT Pace, the watches showed a moderate correlation in regression analysis (r = 0.73–0.88), but the actual prediction accuracy was not high. Therefore, although the smartwatches demonstrated acceptable average accuracy in estimating LT HR, the wide individual variability and limits of agreement suggest that their outputs may still lead to misclassification of physiological training zones, particularly near threshold boundaries (Iannetta et al., 2018). For LT Pace, a systematic overestimation was observed, which further contributes to the risk of distorted intensity guidance. For runners who require precise intensity control, such inaccuracies could result in excessive training loads or insufficient recovery, potentially impairing long-term performance and increasing the risk of injury or overtraining. While smartwatches offer convenience and the ability to monitor general training trends, their outputs should be interpreted with caution when applied to individualized training programs. Given the potential for zone boundary distortion and physiological variability (Iannetta et al., 2023), relying solely on device-generated thresholds may result in inappropriate intensity prescription. Athletes and recreational runners alike are advised to complement wearable data with subjective indicators, ensuring a more holistic and adaptive approach to training regulation.

### 4.5 Limitation

The present study encounters some challenges with variability in data samples among different watches. First, the data for the Coros® watch, although providing valuable insights, is relatively minor compared to those for other watches, which might influence the extrapolation of the findings. Second, a portion of participants experienced smartwatch test failures, in which the device failed to generate LT HR or LT Pace values despite completing the test. This may have influenced the accuracy and completeness of the results. Third, the study measures runners’ LTs using an indoor incremental load test, whereas the watch tests were conducted outdoors. Environmental variables could have impacted the results. Additionally, short-term physiological fluctuations in participants may have contributed to day-to-day variability in LT. Fourth, the devices differed in the methods of HR acquisition during LT testing. Huawei® and Coros® relied on optical PPG sensors, while Garmin® used an ECG-based chest strap. LT estimation depends on accurate HR and speed dynamics. PPG sensors are more susceptible to motion artifacts, which may have affected detection performance during running. This methodological variation could contribute to differences in device accuracy. Future studies should standardize testing protocols, harmonize heart rate monitoring methods, control for internal and external sources of variability, and ensure balanced sample sizes for each device.

## Data Availability

The raw data supporting the conclusions of this article will be made available by the authors, without undue reservation.
