# Correction: Validity of smartwatch-derived estimates of lactate threshold heart rate and pace compared to graded exercise testing

**DOI:** 10.3389/fphys.2025.1661421

**Published:** 2025-07-16

**Authors:** Changda Lu, Wei Cui, Zheng Zhu, Yiwei Wu, Qingjun Xing, Bingyu Pan, Yanfei Shen

**Affiliations:** ^1^School of Sport Science, Beijing Sport University, Beijing, China; ^2^School of Sports Engineering, Beijing Sport University, Beijing, China; ^3^China Sports Big Data Center, Beijing Sport University, Beijing, China; ^4^Engineering Research Center of Strength and Conditioning Training Key Core Technology Integrated System and Equipment, Ministry of Education, Beijing, China

**Keywords:** exercise intensity, running, lactate threshold, smartwatch, validity

In the published article, there was a mistake in the **Corresponding author** designation. Author Yanfei Shen was erroneously omitted as the corresponding author in the published article. The correct corresponding authors appear below.

“Bingyu Pan, panbingyu@bsu.edu.cn; Yanfei Shen, syf@bsu.edu.cn.”

The original article has been updated.

